# Pan-cancer analysis of *MET* mutation and its association with the efficacy of immune checkpoint blockade

**DOI:** 10.1016/j.gendis.2024.101450

**Published:** 2024-11-08

**Authors:** Lijin Chen, Yingying Li, Hong Zhao, Jinyuan Huang, Huimeng Yan, Xiaoyan Lin, Bin Zhao

**Affiliations:** a Fujian Medical University Union Hospital, Fuzhou, Fujian 350001, China; b Quanzhou First Hospital Affiliated to Fujian Medical University, Quanzhou, Fujian 362000, China; c The Cancer Center of The Fifth Affiliated Hospital of Sun Yat-Sen University, Zhuhai, Guangdong 519000, China

**Keywords:** Biomarker, Cancer, Immune checkpoint inhibitor, Immunotherapy, Mesenchymal-epithelial transition factor, Tumor immunogenicity

## Abstract

The mesenchymal-epithelial transition factor (*MET*) proto-oncogene plays important roles during tumor development. Recently, evidence has revealed *MET* signaling may impact tumor immunogenicity and regulate the immune response. Here we conducted a comprehensive bioinformatic and clinical analysis to explore the characteristics of *MET* mutation and its association with the outcomes in pan-cancer immunotherapy. In 4149 patients with 12 tumor types treated with immune checkpoint inhibitors, *MET* mutation indicated favorable overall survival (hazard ratio = 0.61; 95% CI, 0.50–0.74; *P* < 0.001), progression-free survival (hazard ratio = 0.74; 95% CI, 0.60–0.92; *P* = 0.01), and objective response rate (40.3% *vs*. 28.1%; *P* = 0.003). Moreover, we developed a nomogram to estimate the 12-month and 24-month survival probabilities after the initiation of immunotherapy. Further multi-omics analysis on both intrinsic and extrinsic immune landscapes revealed that *MET* mutation enhanced tumor immunogenicity, enriched infiltration of immune cells, and improved immune responses. In summary, *MET* mutation improves cancer immunity and is an independent biomarker for favorable outcomes in pan-cancer immunotherapy. These results may influence clinical practices, guide treatment decision-making, and develop immunotherapy for personalized care.

## Introduction

The application of immunotherapy in clinical practice has revolutionized cancer treatment since 2011.^1^ Immune checkpoint inhibitors targeting programmed cell death ligand 1 (PD-L1), programmed cell death protein 1 (PD-1), and cytotoxic T-lymphocyte-associated antigen 4 (CTLA-4) can significantly improve the outcomes and emerge as standard treatments in many tumors.^2^^,^^3^ However, most cancer patients cannot benefit from immune checkpoint inhibitors.^2^ Although several biomarkers including PD-L1 expression, tumor mutation burden, and microsatellite instability-high/deficient DNA mismatch repair have been granted in the past several years, currently, it is still difficult to determine which patients should be offered immunotherapy.^2^^,^^3^

The *mesenchymal-epithelial transition factor (MET)* proto-oncogene encodes the tyrosine kinase receptor of the hepatocyte growth factor (HGF) and elicits various intracellular signaling cascades including cell motility, growth and survival pathways such as extracellular signal-regulated kinase 1 (ERK1)/mitogen-activated protein kinase (MAPK) and phosphoinositide 3-kinase (PI3K)/protein kinase B (AKT) and inflammation pathways such as signal transducer and activator of transcription (STAT) and nuclear factor kappa B (NF-κB).^4^ These pathways can further mediate physiologic processes including embryogenesis, wound healing, tissue regeneration, organ development, and angiogenesis.^4^ In cancer, aberrant or deregulated MET signaling promotes cell proliferation and survival, sustaining the onset and progression of cancer.^4^^,^^5^ Moreover, MET is a key player in promoting invasive growth and is considered a crucial oncogene in cancer development.^4^^,^^5^ Indeed, the alteration of *MET* is known as one of the most important activated oncogenes and has been a focus since the beginning of cancer-targeted therapy.^5^ Recently, accumulating evidence has revealed that *MET* signaling is also involved in immune response. It is reported MET itself is a broadly expressed tumor-associated antigen that can be recognized by CD8^+^ cytotoxic T-cells.^6^ Additionally, both *in vivo* and *in vitro* studies revealed that the alteration of *MET* could modulate the function of dendritic cells through *HGF/MET* signaling,^7^ the release of cytokines including transforming growth factor-beta (TGF-β), interferon-gamma (IFN-γ), interleukin-10 (IL-10), and tumor necrosis factor-alpha (TNF-α),^8^ and IFN-γ-induced *PD-L1* expression by Janus kinase (*JAK*)*/STAT3* pathway.^9^

Since *MET* signaling appears to play a significant role in immune suppression, we speculated that the mutation of *MET* could impact the efficacy of immunotherapy and come to be a potential predictive biomarker. Due to the rarity of *MET*-mutant tumors, a pooled analysis of available data may provide critical and meaningful information that is helpful in clinical practice. Accordingly, with accumulating data available, here we conducted a comprehensive bioinformatic and clinical analysis to explore the characteristics of *MET* mutation and its association with the outcomes in pan-cancer immunotherapy.

## Material and methods

### Data collection for analyzing tumor immune microenvironment and cancer immunotherapy

Information regarding *MET* alteration and clinicopathological characteristics in patients treated with immune checkpoint inhibitors were collected from 15 published cohorts (Table 1). MSK-IMPACT panel, an integrated genomic profiling panel approved by the US FDA, was applied in one cohort.^10^ Foundation One T7 assay was conducted in a renal cancer cohort.^11^ Whole-exome sequencing was employed for sequencing in the rest cohorts. Gene alteration, RNA expression, and clinical features of 10,953 patients with 33 types of tumors in The Cancer Genome Atlas (TCGA) cohort were downloaded from https://gdc.cancer.gov/about-data/publications/pancanatlas.

**Table 1:** Baseline characteristics of the eligible trials included in this study.

Author, year	Treatment agents	Cancer type	Detection method	*MET* mutation	Cases (*n*)	Response cases (*n*)
*Discovery cohort*						
Hugo, 2016^23^	Pembrolizumab/nivolumab	Melanoma	Whole-exome sequencing	Positive	7	5
				Negative	31	16
Liu, 2019^24^	Pembrolizumab/nivolumab	Melanoma	Whole-exome sequencing	Positive	22	12
				Negative	122	43
Miao, 2018^25^	Inhibitors targeting CTLA-4, PD-1, and PD-L1	Multiple tumors	Whole-exome sequencing	Positive	23	9
				Negative	226	61
Riaz, 2017^26^	Nivolumab	Melanoma	Whole-exome sequencing	Positive	5	2
				Negative	63	13
Van Allen, 2015^27^	Ipilimumab	Melanoma	Whole-exome sequencing	Positive	10	1
				Negative	100	16
Gandara, 2018^28^	Atezolizumab	Lung cancer	Whole-exome sequencing	Positive	10	2
				Negative	417	60
Ravi, 2023^29^	Inhibitors targeting CTLA-4 and PD-1 or in combination with chemotherapy	Lung cancer	Whole-exome sequencing	Positive	11	6
				Negative	298	115
Snyder, 2014^30^	Ipilimumab/tremelimumab	Melanoma	Whole-exome sequencing	Positive	5	Not available
				Negative	59	Not available
Hellmann, 2018^31^	Inhibitors targeting CTLA-4 and PD-1	Lung cancer	Whole-exome sequencing	Positive	5	2
				Negative	70	22
Miao, 2018^32^	Nivolumab	Renal cell carcinoma	Whole-exome sequencing	Positive	3	0
				Negative	32	7
Motzer, 2020^11^	Atezolizumab + bevacizumab	Renal cell carcinoma	Foundation One assay	Positive	19	8
				Negative	340	131
Motzer, 2020^33^	Avelumab + axitinib	Renal cell carcinoma	Whole-exome sequencing	Positive	17	Not available
				Negative	349	Not available
Rizvi, 2015^34^	Pembrolizumab	Lung cancer	Whole-exome sequencing	Positive	2	1
				Negative	32	11
Braun, 2020^35^	Nivolumab	Renal cell carcinoma	Whole-exome sequencing	Positive	6	1
				Negative	255	55
*Validation cohort*						
Samstein, 2019^10^	Inhibitors targeting CTLA-4, PD-1, and PD-L1	Multiple tumors	MSK-IMPACT panel	Positive	61	3
				Negative	1549	49

Note: PD-L1, programmed cell death ligand 1; PD-1, programmed cell death protein 1; CTLA-4, cytotoxic T-lymphocyte-associated antigen 4.

Information regarding overall response rate, progression-free survival, and overall survival were collected from study investigators and relevant repositories. Briefly, responses were defined according to Response Evaluation Criteria in Solid Tumors (RECIST) version 1.1. Patients who showed complete response or partial response were categorized as responders; patients who experienced stable disease or progressive disease were classified as non-responders. Overall survival was defined as the time from the date of first immunotherapy to the time of death from any cause or last follow-up. Progression-free survival was referred to the time from the date of first immunotherapy until the first clinical or radiological progression or death from any cause, whichever occurred first.

Key characteristics related to immunotherapy (non-silent mutation rate, silent mutation rate, indel neoantigen, single nucleotide variant neoantigen, leukocyte fraction, lymphocyte fraction, tumor-infiltrating lymphocyte regional fraction, CD8 T cell abundance, B or T cell receptor richness, and B or T cell receptor Shannon index) were calculated as previously described.^12^^,^^13^ The enrichment levels of 29 classical immune signatures were evaluated according to the single-sample gene set enrichment analysis (ssGSEA) method using the “GSVA” R package.^14^ The MCP-counter approach was introduced to estimate the population abundance of tissue-infiltrating eight immune (CD8^+^ T cells, CD3^+^ T cells, cytotoxic lymphocytes, B lymphocytes, natural killer cells, cells originating from monocytes, neutrophils, and myeloid dendritic cells) and two stromal cell populations (endothelial cells and fibroblasts).^15^

### Deciphering mutational signatures

The “deconstructSigs” R package was applied to perform a non-negative matrix factorization analysis of mutations and patterns of carcinoma evolution.^16^ The extracted mutation pattern was compared against the COSMIC based on cosine similarity.

### Generation and validation of the nomogram

Nomograms have been widely used in oncology to predict outcomes quantitatively using critical predictive features. A calibration curve could be used to evaluate the similarity between the predictive and actual survival probability. The “rms” R package was used to generate both nomogram and calibration curves.

### Statistics

Survival curves were generated by the Kaplan-Meier method to reflect the differences in survival and the log-rank test was used to evaluate the statistical significance of differences. The hazard ratio (HR) was calculated by the Cox proportional hazards model and a 95% confidence interval (CI) was reported. Kruskal-Wallis, Wilcoxon test, and Chi-square test were used to analyze the associations among various categorical variables depending on the context. All data processing and analysis were performed with R software (version 4.2.1). Two-sided *P* values < 0.05 were considered statistically significant.

## Results

**Figure 1:**
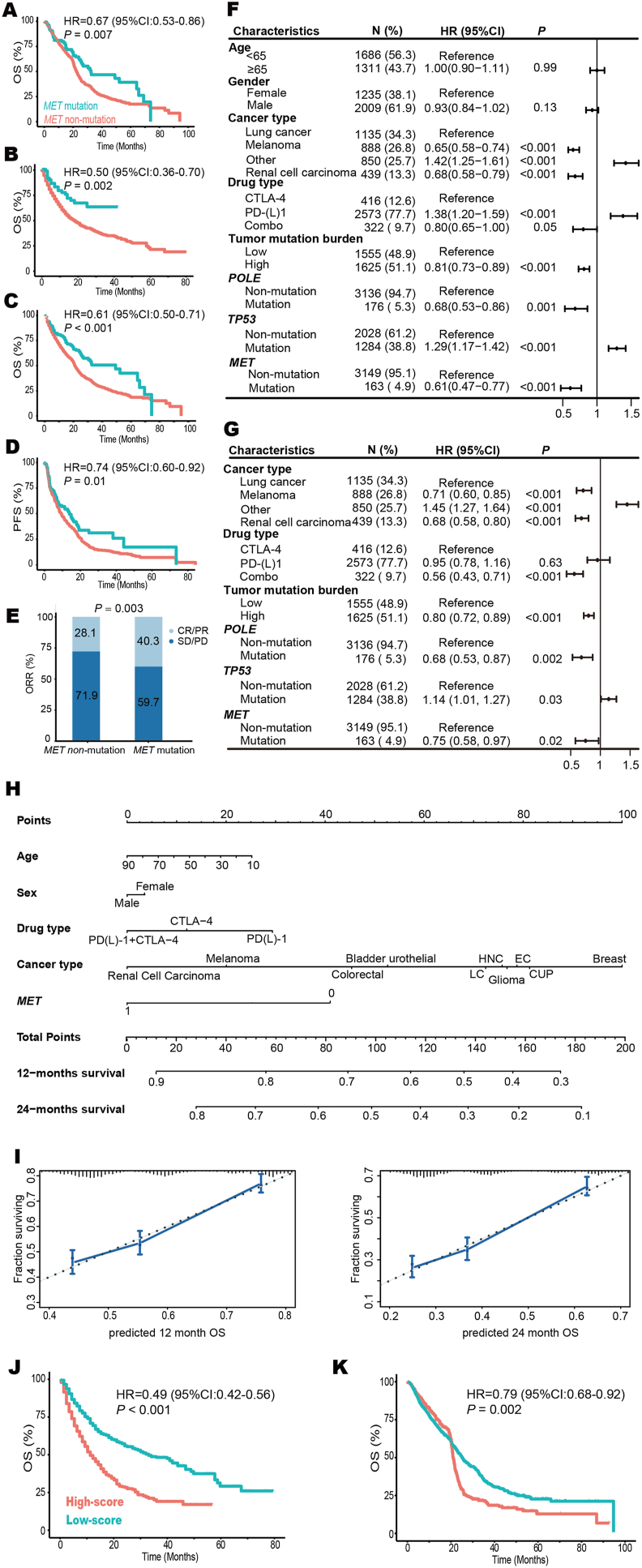
MET mutation as an independent biomarker for favorable outcomes in pan-cancer immunotherapy. **(A)** Kaplan–Meier survival analysis stratified by *MET* mutation status in 2539 cancer patients with 7 tumor types treated with ICIs in the discovery cohort. **(B)** The association between *MET* mutation and OS in 1610 patients with 10 tumor types in the validation cohort. **(C**–**E)** The comparison of OS (C), PFS (D), and ORR (E) between patients with *MET* mutation and patients with *MET* non-mutation in 4149 subjects with 12 tumors treated with ICIs. **(F, G)** Univariate (F) and multivariate (G) Cox analyses of the association between *MET* mutation and OS in 4149 patients with 12 tumors treated with ICIs. **(H)** The nomogram for predicting the 12- and 24-month survival. It can calculate overall survival from the date of immunotherapy start. To use, users should locate the “age” axis and draw a line up to the “point” axis to get a score associated with age and repeat for the other features to get their scores. Afterward, the users sum all scores, locate it on the “total point” axis, and draw a line to the “12-month survival” axis to get the 12-month OS probability. **(I)** Calibration plots for validation of the 12- and 24-month survival from the nomogram in the discovery cohort. The average predicted probability (X-axis) was plotted against the observed Kaplan-Meier estimate in the subgroup (Y-axis, 95% CIs of the estimates are presented as vertical lines). The continuous line is the reference line, indicating what an optimal nomogram would be. **(J, K)** Based on the optimal cutoff value (total points = 60) derived from the nomogram, a low score was associated with favorable OS in both the discovery cohort (J) and validation cohort (K). CI, confidence interval; CUP, cancer of unknown primary; CR, complete response; EC, esophagogastric cancer; HNC, head and neck carcinoma; HR, hazard ratio; ICI, immune checkpoint inhibitor; LC, lung cancer; ORR, objective response rate; OS, overall survival; PFS, progression-free survival; PD, progressive disease; PR, partial response; SD, stable disease.

To investigate the impact of *MET* mutation on the efficacy of immunotherapy, 2539 patients with 7 tumor types from 14 datasets were applied as a discovery cohort (Table 1). These subjects were diagnosed with lung cancer (*n* = 902), renal cell carcinoma (*n* = 1021), melanoma (*n* = 575), bladder urothelial cancer (*n* = 27), head and neck cancer (*n* = 12), sarcoma (*n* = 1), and anal cancer (*n* = 1). Totally, *MET*-mutant tumors were identified in 145 patients and were associated with longer overall survival (HR = 0.67; 95% CI, 0.53–0.86; *P* = 0.007; Fig. 1A). We further collected 1610 patients with 10 tumor types as a validation cohort, including lung cancer (*n* = 344), melanoma (*n* = 314), bladder urothelial cancer (*n* = 211), renal cell carcinoma (*n* = 143), head and neck cancer (*n* = 129), esophagogastric cancer (*n* = 118), glioma (*n* = 116), colorectal cancer (*n* = 109), cancer of unknown primary (*n* = 85), and breast cancer (*n* = 41). Sixty-one patients with *MET*-mutant tumors also achieved favorable outcomes (HR = 0.50; 95% CI, 0.36–0.70; *P* = 0.002; Fig. 1B). Total, in 4149 patients with 12 tumor types treated with immune checkpoint inhibitors, *MET* mutation (*n* = 206) decreased the risk of death by 39% (HR = 0.61; 95% CI, 0.50–0.74; *P* < 0.001; Fig. 1C). Additionally, patients with *MET* mutation showed better progression-free survival (HR = 0.74; 95% CI, 0.60–0.92; *P* = 0.01; Fig. 1D) and overall response rate (40.3% *vs*. 28.1%; *P* = 0.003; Fig. 1E).

**Figure S1:**
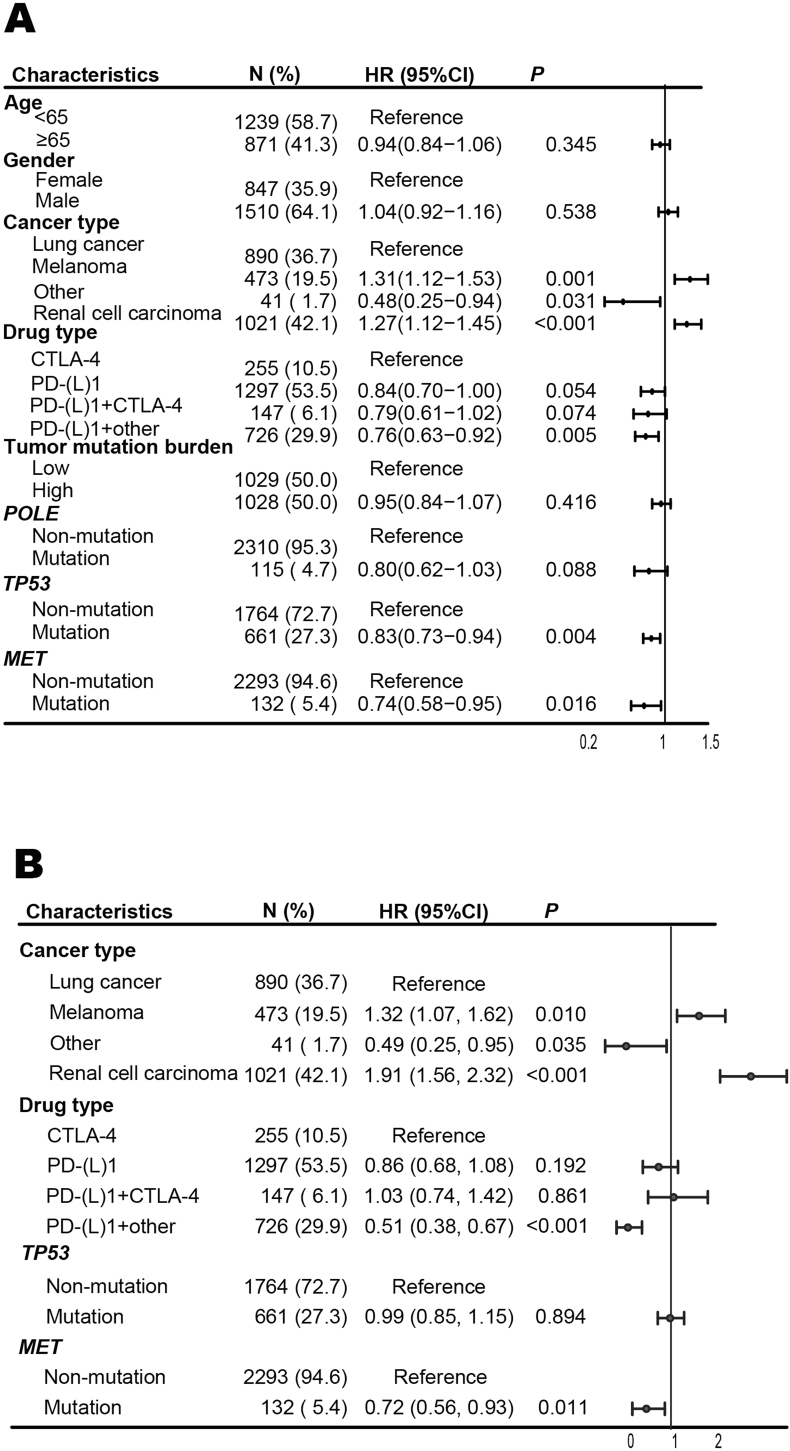
Univariate **(A)** and multivariate **(B)** Cox analyses of the association between *MET* mutation and progression-free survival in 4149 patients with 12 tumors treated with immune checkpoint inhibitors.

To evaluate the performance of various characteristics as biomarkers for overall survival in patients treated with immune checkpoint inhibitors, we conducted univariate (Fig. 1F) and multivariate (Fig. 1G) Cox analyses. *MET* mutation was an independent favorable predictor for overall survival (HR = 0.75; 95% CI, 0.58–0.97; *P* = 0.02). As expected, similar results were observed in progression-free survival multivariate Cox analysis (HR = 0.72; 95% CI, 0.56–0.93; *P* = 0.01; Fig. S1). Then we constructed a nomogram to estimate the 12-month and 24-month survival based on the discovery cohort (Fig. 1H). Further examination of the calibrations for these predictions revealed the performance of this cure-model-based nomogram was good (Fig. 1I), with the potential to estimate the survival probabilities after the initiation of immunotherapy in clinical practice. Moreover, we applied X-tile software to determine the optimal cutoff value (total points = 118) and classified all the enrolled patients into low- and high-score subgroups. A low score was associated with longer overall survival in both the discovery cohort (HR = 0.49; 95% CI, 0.42–0.56; *P* < 0.001; Fig. 1J) and validation cohort (HR = 0.79; 95% CI, 0.68–0.92; *P* = 0.002; Fig. 1K).

**Figure 2:**
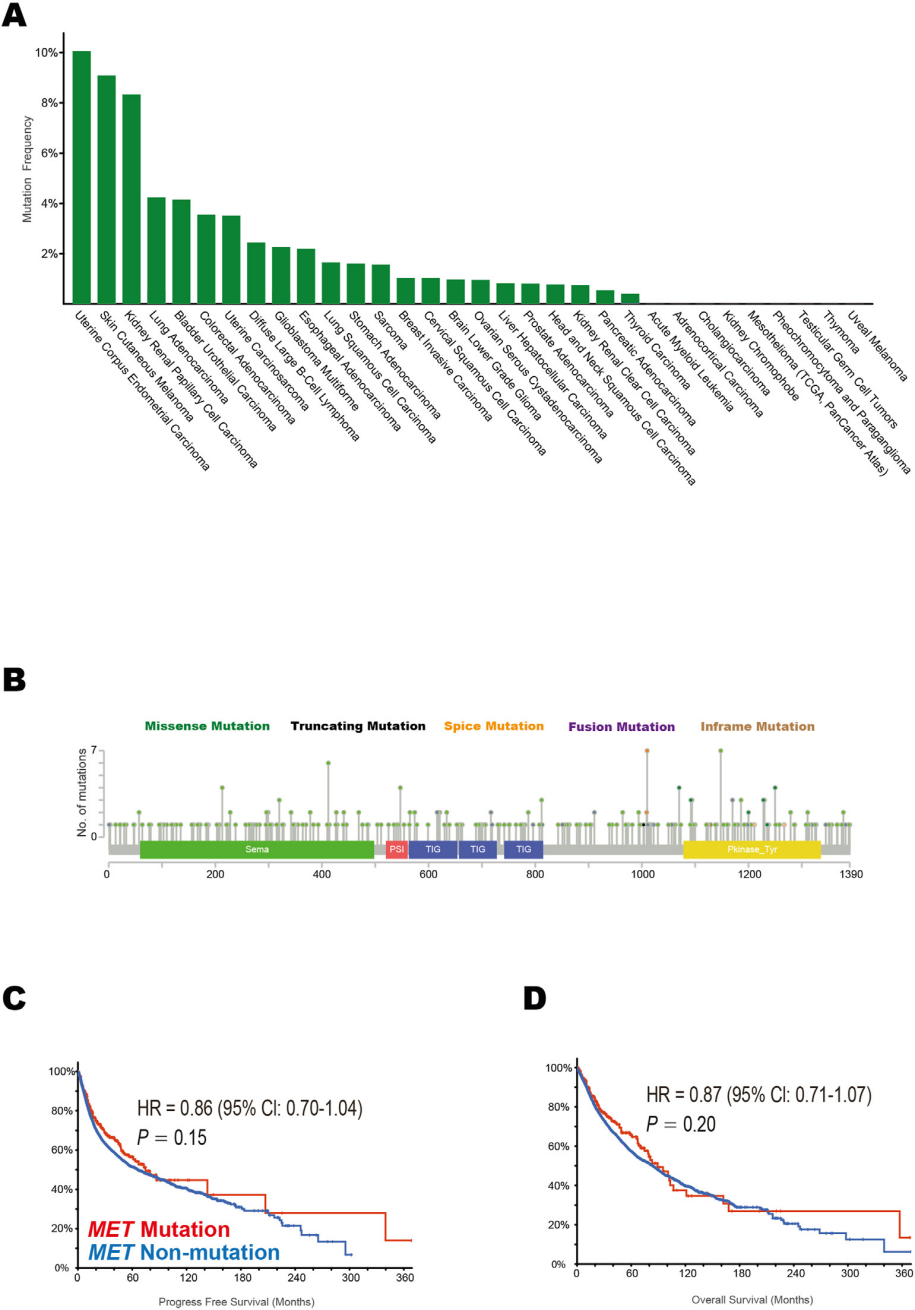
The characteristics of *MET* mutation in 33 tumor types based on The Cancer Genome Atlas (TCGA) cohort. **(A)** The mutation frequencies of *MET* gene across 33 tumor types. **(B)** The subtypes and distributions of *MET* somatic mutations. X-axis, amino acid; Y-axis, numbers of *MET* mutations. Sema, Sema domain (59–498); PSI, plexin repeat (520–561); PSI, plexin repeat (520–561; 657–728; 742–815); Pkinase_Tyr, protein tyrosine kinase (1078–1336). Green, missense mutation; black, truncating mutation; orange, splice mutation; purple, fusion mutation; brown, Inframe mutation. **(C, D)** Comparison of PFS (C) and OS (D) between patients with *MET* mutation and patients with *MET* non-mutation in 10,953 subjects with 33 tumor types. PFS, progression-free survival; OS, overall survival.

To investigate the underlying mechanisms between cancer immunotherapy and *MET* mutation, multi-omics information extracted from the TCGA pan-cancer cohort was explored. We first examined the somatic mutant frequencies of *MET* and discovered that 251 of all 10,953 enrolled patients (2.29%) harbored *MET* mutations. *MET* mutations were found in most tumor types (Fig. 2A), and the mutant frequencies differed significantly among various tumors (*P* < 0.001). In total, 311 mutations were identified, 250 (80.39%) were missense mutations, 32 (10.29%) were truncating mutations, 15 (4.82%) were splice mutations, 13 (4.18%) were fusion mutations, and 1 (0.32%) was inframe mutation. These mutations occurred in a dispersed manner throughout the whole sequence (Fig. 2B). Further analysis revealed that the prognosis for cancer patients was independent of *MET* mutations in terms of progression-free survival (HR = 0.86; 95% CI, 0.70–1.04; *P* = 0.15; Fig. 2C) and overall survival (HR = 0.87; 95% CI, 0.71–1.07; *P* = 0.20; Fig. 2D).

**Figure 3:**
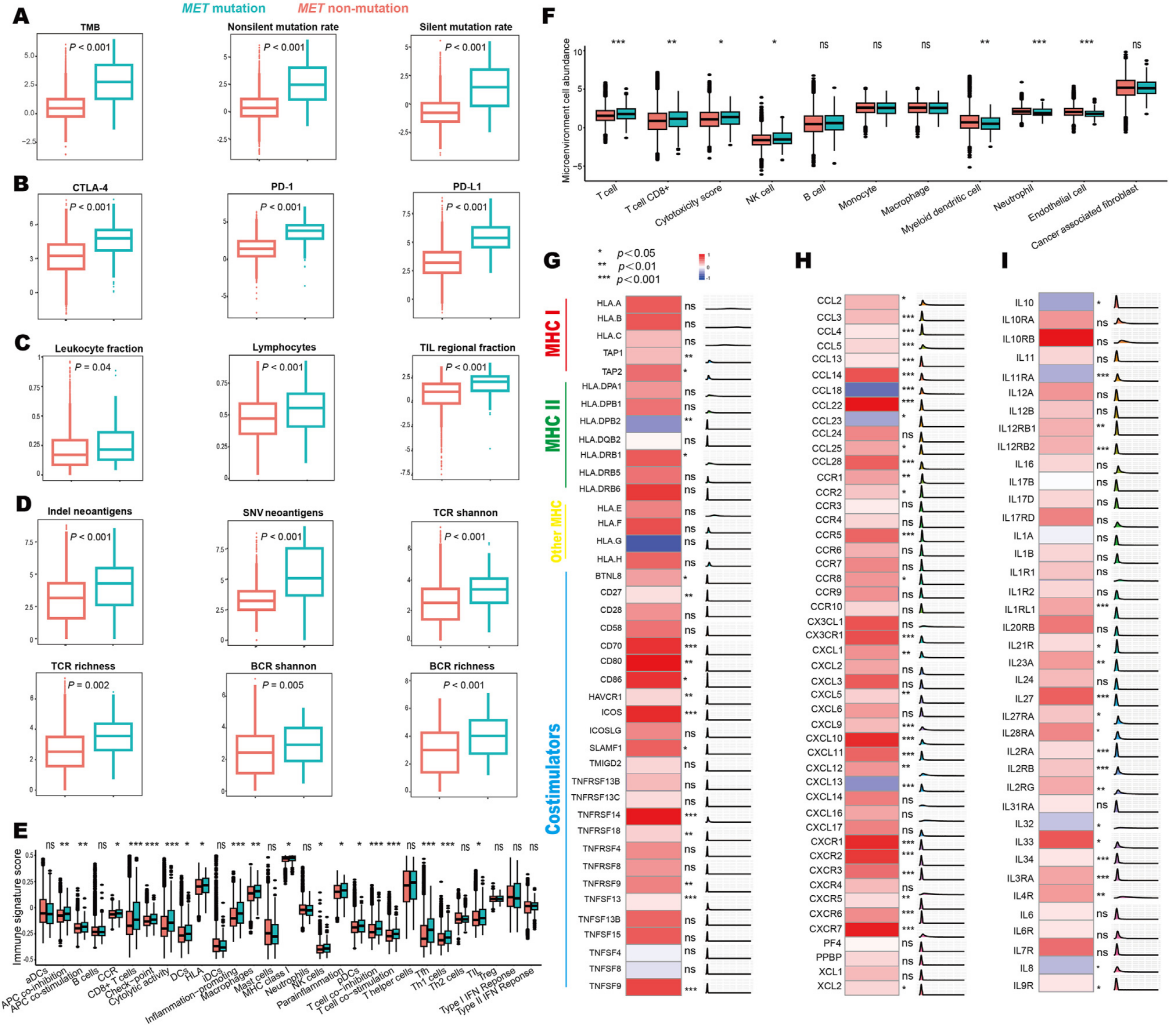
The differences in tumor immune microenvironment between patients with *MET-*mutant and *MET*-non-mutant tumors. **(A)** Comparison of TMB, non-silent mutation rate, and silent mutation rate between *MET-*mutant and *MET*-non-mutant tumors. **(B)** mRNA expression levels of PD-1, PD-L1, and CTLA-4 in patients with *MET-*mutant and *MET-*non-mutant tumors. **(C)** The immune cell infiltration revealed by leukocyte fractions, lymphocyte fraction, and tumor-infiltrating lymphocyte fraction in *MET-*mutant and *MET*-non-mutant tumors. **(D)** The abundances of SNV neoantigens/Indel neoantigens and the diversity of TCR/BCR in *MET-*mutant and *MET*-non-mutant tumors. **(E)** Differences of 29 immune signatures estimated by ssGSEA between *MET-*mutant and *MET-*non-mutant tumors. **(F)** Comparison of 8 immune and 2 stromal cell populations between *MET-*mutant and *MET-*non-mutant tumors. **(G)** Expression differences of 16 MHC-related antigen-presenting molecules and 25 co-stimulators between *MET-*mutant and *MET-*non-mutant tumors. **(H)** Comparison of 48 chemokines and their receptors between *MET-*mutant and *MET-*non-mutant tumors. **(I)** Expression differences of 39 immune-stimulators between *MET-*mutant and *MET-*non-mutant tumors. BCR, B cell receptor; CTLA-4, cytotoxic T-lymphocyte-associated antigen 4; MHC, major histocompatibility complex; PD-1, programmed cell death protein 1; PD-L1, programmed cell death ligand 1; SNV, single nucleotide variants; TCR, T cell receptor; TIL, tumor-infiltrating lymphocyte; TMB, tumor mutation burden.

**Figure 4:**
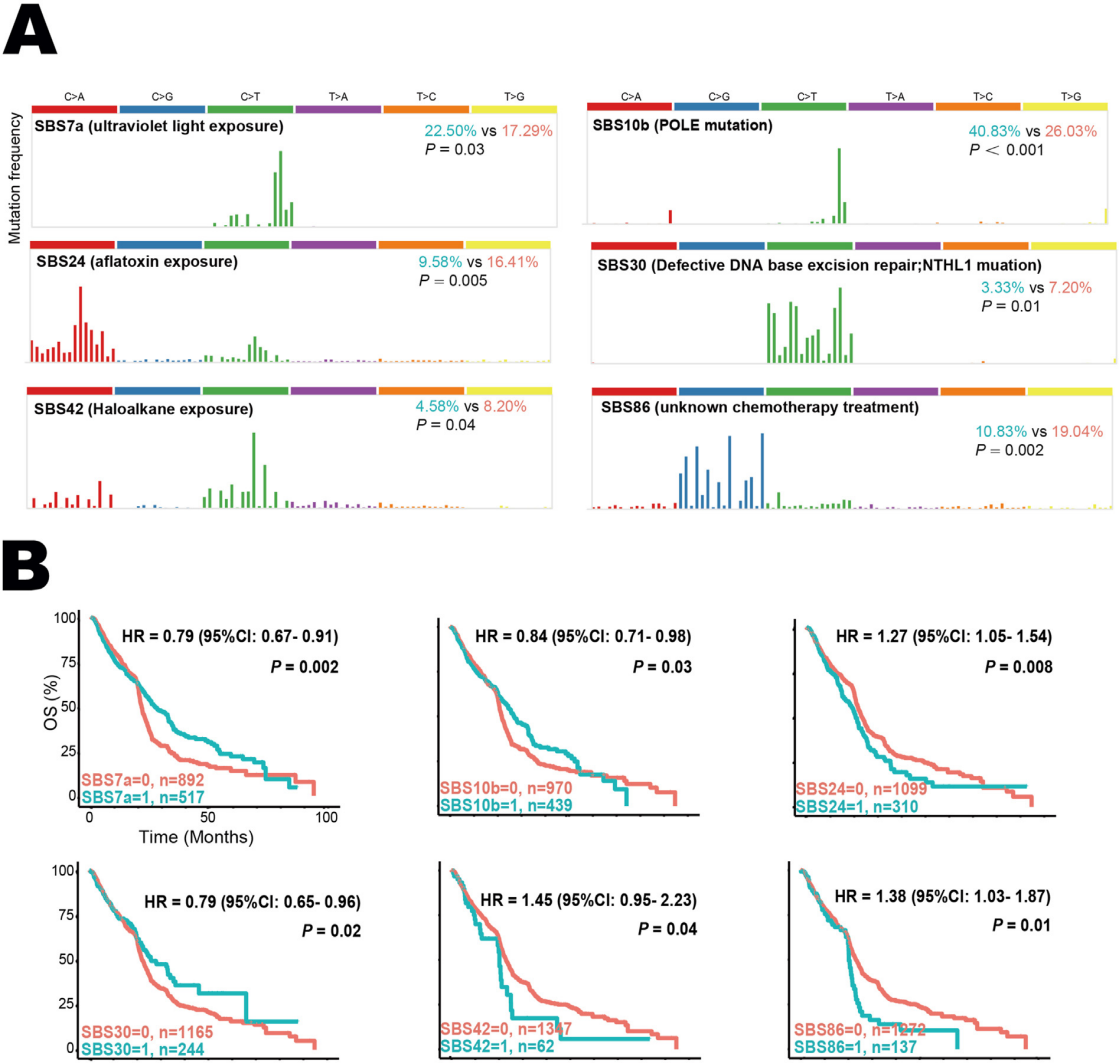
COSMIC reference signatures associated with *MET* mutation. **(A)** The illustrations of four identified SBS signatures related to *MET* mutation and their frequencies in *MET*-mutant and *MET*-non-mutant tumors. Bold black, SBS signature and its known etiologies. Green, frequency in *MET*-mutant cancer. Orange, frequency in *MET*-non-mutant cancer. **(B)** The associations between four identified mutation signatures with OS in cancer immunotherapy. HR, hazard ratio; OS, overall survival; SBS, single base substitution.

The key intrinsic immune response mainly referred to high tumor immunogenicity, activation of the antigen-processing machinery, and over-expression of costimulatory molecules.^17^ The mutation loads including tumor mutation burden, non-silent mutation rate, and silent mutation rate were significantly higher in *MET*-mutant tumors (Fig. 3A). Next, we examined if any specific mutation patterns were associated with the outcomes in *MET*-mutant patients treated with immune checkpoint inhibitors. As shown in Figure 4A, the prevalence of COSMIC reference signatures SBS7a (known etiology, ultraviolet light exposure; 22.50% *vs*. 17.29%; *P* = 0.03), SBS10b (*POLE* mutation; 40.83% *vs*. 26.03%; *P* < 0.001), SBS24 (aflatoxin exposure; 9.58% *vs*. 16.41%; *P* = 0.005), SBS30 (defective DNA base excision repair and *NTHL1* mutation; 3.33% *vs*. 7.20%; *P* = 0.01), SBS42 (Haloalkane exposure; 4.58% *vs*. 8.20%; *P* = 0.04), and SBS86 (unknown chemotherapy; 10.83% *vs*. 19.04%; *P* = 0.002) were significantly different between *MET*-mutant and *MET*-non-mutant tumors. These SBSs were further identified as robust predictive biomarkers for survival in pan-cancer immunotherapy (Fig. 4B). Indeed, the occurrence of SBS7a (HR = 0.79; 95% CI, 0.67–0.91; *P* = 0.002), SBS10b (HR = 0.84; 95% CI, 0.71–0.98; *P* = 0.03), and SBS30 (HR = 0.79; 95% CI, 0.65–0.96; *P* = 0.02) indicted favorable outcomes, while SBS24 (HR = 1.27; 95% CI, 1.05–1.54; *P* = 0.008), SBS42 (HR = 1.45; 95% CI, 0.95–2.23; *P* = 0.04), and SBS86 (HR = 1.38; 95% CI, 1.03–1.87; *P* = 0.01) were negative predictors. Additionally, the expression of three key immune checkpoints (*CTLA-4*, *PD-1*, and *PD-L1*) was significantly increased in *MET*-mutant tumors (Fig. 3B). The dysfunctions of major histocompatibility complex (MHC) were the main cause of tumor immune escape.^2^ *MET* mutation was associated with increased expression of many MHC-related antigen-presenting molecules and co-stimulators (Fig. 3G).

The major extrinsic immune features including the infiltration of immune cells, high diversity of B or T cell receptors, and activated immunogenicity of cancer cells contribute to the immune response and high expression of immune stimulators and chemokines.^18^ *MET* mutations were associated with higher levels of immune cell infiltration according to (i) leukocyte fractions measured by DNA methylation arrays; (ii) lymphocyte fractions estimated from the CIBERSORT algorithm; and (iii) tumor-infiltrating lymphocyte fractions evaluated by RNA-sequencing information (Fig. 3C). Mutations in tumors could induce potential tumor-associated neoantigens, which were recognized by T cells with antigen-specific T cell receptors or B cells with B cell receptors. Compared with *MET*-non-mutant tumors, the abundances of single nucleotide variant neoantigens/Indel neoantigens and the diversity of T or B cell receptors were significantly up-regulated in *MET*-mutant tumors (Fig. 3D). ssGSEA (Fig. 3E), a method that quantifies the expressions of 29 immune signatures, presented the key immune pathways, cells, and functions.^14^ The MCP-counter method calculated the abundance of 8 immune and 2 stromal cell populations (Fig. 3F).^15^ Both approaches revealed that the immune cell populations and immune activities were clearly enriched in *MET*-mutant tumors. It was well-established that patients with immunologically “hot” tumors, characterized by higher levels of CD8^+^ T infiltration, showed favorable outcomes to immunotherapy.^19^ Hence, we specifically examined the abundances of CD8^+^ T cells and found that they were significantly increased in *MET*-mutant tumors estimated by both ssGSEA and MCP-counter methods. Moreover, *MET* mutation was associated with higher levels of most chemokines and their receptors (Fig. 3H) and immune-stimulators (Fig. 3I).

## Discussion

It is well-established that actionable drivers have re-defined cancer treatment and become a key element during therapeutic decision-making. Meanwhile, these oncogenic drivers can also impact the systemic and local immune landscape, and their roles in immunotherapy are incompletely understood.^20^ Here, 4149 patients with 12 tumor types were pooled to systematically evaluate the impact of *MET* mutation on the efficacy of immune checkpoint inhibitors. Our data revealed that *MET* mutation indicated favorable outcomes in pan-cancer immunotherapy. Moreover, we developed a nomogram to estimate the 12-month and 24-month survival after the initiation of immunotherapy in real-world clinical practice. Further multi-omics analysis based on the TCGA cohort revealed *MET* mutation was associated with enriched infiltration of immune cells, enhanced tumor immunogenicity, and improved immune responses. These findings may influence clinical practices, guide decision-making, develop immunotherapy for personalized care, and ultimately improve patient outcomes.

The exact underlying molecular mechanisms between *MET* mutation and tumor immune microenvironment were largely unknown, but the intricate role of HGF/MET was multifaceted in cancer. First, MET itself could elicit immune system activation against cancer cells overexpressing MET through recognition by CD8^+^ cytotoxic T-cells.^6^ It was also reported that after the stimulation of the HGF/MET signaling pathway, renal cancer cells exhibited the up-regulation of PD-L1 expression through the PI3K pathway.^21^ Second, MET played an important role in regulating the function of dendritic cells, which were responsible for presenting tumor-associated antigens and activating regulatory CD4^+^ T cells that control cytotoxic CD8^+^ T cells. Studies have indicated that HGF/MET could enhance this function, suggesting a beneficial impact on anti-cancer immunity.^7^ However, conflicting evidence exists, as some studies have shown HGF/MET might also act as a potent inhibitor of dendritic cell function, leading to an increase in regulatory T lymphocytes, reduction in interleukin-17 (IL-17)-producing lymphocytes, coupled with elevation of IL-10 and TGF-β, indicating a suppression of the immune response.^8^ This inhibitory impact could extend to CD8^+^ T lymphocytes, regulatory T cells, and monocytes. Third, besides antigen-presenting cells, the interaction between HGF/MET and the immune system was also evident in granulocytes. Notably, *MET* was essential for neutrophil-mediated cytotoxicity, as deletion of *MET* in neutrophils had been shown to promote tumor growth and metastasis.^22^ This phenomenon was further supported by clinical data showing a correlation between *MET* deletion and decreased neutrophil infiltration in both primary tumors and distant metastases. In this study, we examined all the major lymphocyte types, their secreted chemokines and receptors, and immune-stimulators. Consistent with previous reports,^8^ most of these features were significantly increased in *MET*-mutant tumors, suggesting *MET*-mutant tumors were immunologically “hot” tumors in clinical practice.

Our study had several limitations. First, these eligible studies were conducted at various medical centers, the tools used in analyzing sequencing data among these studies were different, and the researchers had subjectivities in recording clinical outcomes, especially overall response rate and progression-free survival. Our result was subject to any biases or errors derived from the original investigators. Second, due to the limited number of patients available, we conducted our study based on 12 major tumor types. Hence, we cannot estimate the performance of *MET*-mutant as a prediction biomarker in other tumors. Third, through TCGA cohort analysis, here we discovered the robust association of *MET* mutation with enriched infiltration of immune cells, enhanced tumor immunogenicity, and improved immune responses. Further molecular experiments were needed to explore the exact underlying mechanisms between *MET* mutation and tumor immune microenvironment.

Despite these limitations, with individual patient data derived from over 4000 subjects with 12 major tumor types treated with immune checkpoint inhibitors, this is the first study to systematically examine the performance of *MET* mutation as a predictive biomarker in cancer immunotherapy.

In summary, our results from both extrinsic and intrinsic immune landscapes revealed *MET* mutation was associated with enhanced tumor immunogenicity, enriched infiltration of immune cells, and improved immune responses. Moreover, *MET* mutation was an independent biomarker for favorable outcomes in cancer immunotherapy. This study may influence clinical practices, guide treatment decision-making, and develop immunotherapy for personalized care.

## Funding

This study was supported by the 10.13039/501100001809National Natural Science Foundation of China (No. 82373367).

## CRediT authorship contribution statement

**Lijin Chen:** Data curation, Investigation, Methodology, Validation, Visualization, Writing – original draft, Writing – review & editing. **Yingying Li:** Conceptualization, Formal analysis, Investigation, Methodology, Validation, Visualization, Writing – review & editing. **Hong Zhao:** Conceptualization, Funding acquisition, Writing – review & editing. **Jinyuan Huang:** Investigation, Methodology, Validation, Visualization. **Huimeng Yan:** Investigation, Methodology, Software, Visualization. **Xiaoyan Lin:** Investigation, Supervision, Writing – original draft, Writing – review & editing. **Bin Zhao:** Conceptualization, Supervision, Writing – original draft, Writing – review & editing.

## Data availability

The datasets generated during and/or analyzed during the current study are available from the corresponding author upon reasonable request.

## Conflict of interests

All authors claimed no competing interests.
